# Supplementary material for: A Critical View of the Application of the APEX Software (Aqueous Photochemistry of Environmentally-Occurring Xenobiotics) to Predict Photoreaction Kinetics in Surface Freshwaters
Source: Molecules. 2019 Dec 18;25(1):9. doi: 10.3390/molecules25010009 (PMC7017383; doi:10.3390/molecules25010009)
Supplement: Supplementary file 1 [file molecules-25-00009-s001.zip › 3.2.4_gcc-4.4.0.zip!/3.2.4_gcc-4.4.0/3.2.4_gcc-4.4.0/doc/octave/html/interpreter/Three_002dDimensional-Plotting.html]

Three-Dimensional Plotting - Untitled


Next: Plot Annotations,
Previous: Two-Dimensional Plots,
Up: Plotting Basics


---

#### 15.1.2 Three-Dimensional Plotting

The function `mesh` produces mesh surface plots. For example,

```
     tx = ty = linspace (-8, 8, 41)';
     [xx, yy] = meshgrid (tx, ty);
     r = sqrt (xx .^ 2 + yy .^ 2) + eps;
     tz = sin (r) ./ r;
     mesh (tx, ty, tz);
```

produces the familiar “sombrero” plot shown in fig:mesh. Note
the use of the function `meshgrid` to create matrices of X and Y
coordinates to use for plotting the Z data. The `ndgrid` function
is similar to `meshgrid`, but works for N-dimensional matrices.

**Figure 15.5: Mesh plot.**

The `meshc` function is similar to `mesh`, but also produces a
plot of contours for the surface.

The `plot3` function displays arbitrary three-dimensional data,
without requiring it to form a surface. For example

```
     t = 0:0.1:10*pi;
     r = linspace (0, 1, numel (t));
     z = linspace (0, 1, numel (t));
     plot3 (r.*sin(t), r.*cos(t), z);
```

displays the spiral in three dimensions shown in fig:plot3.

**Figure 15.6: Three dimensional spiral.**

Finally, the `view` function changes the viewpoint for
three-dimensional plots.

— Function File: **mesh** (x, y, z)  
> Plot a mesh given matrices x, and y from `meshgrid` and
> a matrix z corresponding to the x and y coordinates of
> the mesh. If x and y are vectors, then a typical vertex
> is (x(j), y(i), z(i,j)). Thus, columns of z
> correspond to different x values and rows of z correspond
> to different y values.
>
>
>
> **See also:** meshgrid, contour.

— Function File: **meshc** (x, y, z)  
> Plot a mesh and contour given matrices x, and y from
> `meshgrid` and a matrix z corresponding to the x and
> y coordinates of the mesh. If x and y are vectors,
> then a typical vertex is (x(j), y(i), z(i,j)). Thus,
> columns of z correspond to different x values and rows of
> z correspond to different y values.
>
>
>
> **See also:** meshgrid, mesh, contour.

— Function File: **meshz** (x, y, z)  
> Plot a curtain mesh given matrices x, and y from
> `meshgrid` and a matrix z corresponding to the x and
> y coordinates of the mesh. If x and y are vectors,
> then a typical vertex is (x(j), y(i), z(i,j)). Thus,
> columns of z correspond to different x values and rows of
> z correspond to different y values.
>
>
>
> **See also:** meshgrid, mesh, contour.

— Function File: **hidden** (mode)  
— Function File: **hidden** ()  
> Manipulation the mesh hidden line removal. Called with no argument
> the hidden line removal is toggled. The argument mode can be either
> 'on' or 'off' and the set of the hidden line removal is set accordingly.
>
>
>
> **See also:** mesh, meshc, surf.

— Function File: **surf** (x, y, z)  
> Plot a surface given matrices x, and y from `meshgrid` and
> a matrix z corresponding to the x and y coordinates of
> the mesh. If x and y are vectors, then a typical vertex
> is (x(j), y(i), z(i,j)). Thus, columns of z
> correspond to different x values and rows of z correspond
> to different y values.
>
>
>
> **See also:** mesh, surface.

— Function File: **surfc** (x, y, z)  
> Plot a surface and contour given matrices x, and y from
> `meshgrid` and a matrix z corresponding to the x and
> y coordinates of the mesh. If x and y are vectors,
> then a typical vertex is (x(j), y(i), z(i,j)). Thus,
> columns of z correspond to different x values and rows of
> z correspond to different y values.
>
>
>
> **See also:** meshgrid, surf, contour.

— Function File: **surfl** (x, y, z)  
— Function File: **surfl** (z)  
— Function File: **surfl** (x, y, z, L)  
— Function File: **surfl** (x, y, z, L, P)  
— Function File: **surfl** (...,"light")  
> Plot a lighted surface given matrices x, and y from `meshgrid` and
> a matrix z corresponding to the x and y coordinates of
> the mesh. If x and y are vectors, then a typical vertex
> is (x(j), y(i), z(i,j)). Thus, columns of z
> correspond to different x values and rows of z correspond
> to different y values.
>
> The light direction can be specified using L. It can be
> given as 2-element vector [azimuth, elevation] in degrees or as 3-element vector [lx, ly, lz].
> The default value is rotated 45° counter-clockwise from the current view.
>
> The material properties of the surface can specified using a 4-element vector
> P = [AM D SP exp] which defaults to
> p = [0.55 0.6 0.4 10].
>
> `"AM" strength of ambient light` `"D" strength of diffuse reflection` `"SP" strength of specular reflection` `"EXP" specular exponent`
>
> The default lighting mode "cdata", changes the cdata property to give the impression
> of a lighted surface. Please note: the alternative "light" mode, which creates a light
> object to illuminate the surface is not implemented (yet).
>
> Example:
>
> ```
>           colormap(bone);
>           surfl(peaks);
>           shading interp;
> ```
>
>
>
>
> **See also:** surf, diffuse, specular, surface.

— Function File: **surfnorm** (x, y, z)  
— Function File: **surfnorm** (z)  
— Function File: [nx, ny, nz] = **surfnorm** (...)  
— Function File: **surfnorm** (h, ...)  
> Find the vectors normal to a meshgridded surface. The meshed gridded
> surface is defined by x, y, and z. If x and
> y are not defined, then it is assumed that they are given by
>
> ```
>           [x, y] = meshgrid (1:size(z, 1),
>                                1:size(z, 2));
> ```
>
> If no return arguments are requested, a surface plot with the normal
> vectors to the surface is plotted. Otherwise the components of the normal
> vectors at the mesh gridded points are returned in nx, ny,
> and nz.
>
> The normal vectors are calculated by taking the cross product of the
> diagonals of each of the quadrilaterals in the meshgrid to find the
> normal vectors of the centers of these quadrilaterals. The four nearest
> normal vectors to the meshgrid points are then averaged to obtain the
> normal to the surface at the meshgridded points.
>
> An example of the use of `surfnorm` is
>
> ```
>           surfnorm (peaks (25));
> ```
>
>
>
>
> **See also:** surf, quiver3.

— Function File: **diffuse** (sx, sy, sz, l)  
> Calculate diffuse reflection strength of a surface defined by the normal
> vector elements sx, sy, sz.
> The light vector can be specified using parameter L. It can be
> given as 2-element vector [azimuth, elevation] in degrees or as 3-element
> vector [lx, ly, lz].
>
>
>
> **See also:** specular, surfl.

— Function File: **specular** (sx, sy, sz, l, v)  
— Function File: **specular** (sx, sy, sz, l, v, se)  
> Calculate specular reflection strength of a surface defined by the normal
> vector elements sx, sy, sz using Phong's approximation.
> The light and view vectors can be specified using parameter L and V respectively.
> Both can be given as 2-element vectors [azimuth, elevation] in degrees or as 3-element
> vector [x, y, z]. An optional 6th argument describes the specular exponent (spread) se.
>
>
>
> **See also:** surfl, diffuse.

— Function File: [xx, yy, zz] = **meshgrid** (x, y, z)  
— Function File: [xx, yy] = **meshgrid** (x, y)  
— Function File: [xx, yy] = **meshgrid** (x)  
> Given vectors of x and y and z coordinates, and
> returning 3 arguments, return three-dimensional arrays corresponding
> to the x, y, and z coordinates of a mesh. When
> returning only 2 arguments, return matrices corresponding to the
> x and y coordinates of a mesh. The rows of xx are
> copies of x, and the columns of yy are copies of y.
> If y is omitted, then it is assumed to be the same as x,
> and z is assumed the same as y.
>
>
>
> **See also:** mesh, contour.

— Function File: [y1, y2, ..., yn] = **ndgrid** (x1, x2, ..., xn)  
— Function File: [y1, y2, ..., yn] = **ndgrid** (x)  
> Given n vectors x1, ... xn, `ndgrid` returns
> n arrays of dimension n. The elements of the i-th output argument
> contains the elements of the vector xi repeated over all
> dimensions different from the i-th dimension. Calling ndgrid with
> only one input argument x is equivalent of calling ndgrid with
> all n input arguments equal to x:
>
> [y1, y2, ..., yn] = ndgrid (x, ..., x)
>
>
>
> **See also:** meshgrid.

— Function File: **plot3** (args)  
> Produce three-dimensional plots. Many different combinations of
> arguments are possible. The simplest form is
>
> ```
>           plot3 (x, y, z)
> ```
>
> in which the arguments are taken to be the vertices of the points to
> be plotted in three dimensions. If all arguments are vectors of the
> same length, then a single continuous line is drawn. If all arguments
> are matrices, then each column of the matrices is treated as a
> separate line. No attempt is made to transpose the arguments to make
> the number of rows match.
>
> If only two arguments are given, as
>
> ```
>           plot3 (x, c)
> ```
>
> the real and imaginary parts of the second argument are used
> as the y and z coordinates, respectively.
>
> If only one argument is given, as
>
> ```
>           plot3 (c)
> ```
>
> the real and imaginary parts of the argument are used as the y
> and z values, and they are plotted versus their index.
>
> Arguments may also be given in groups of three as
>
> ```
>           plot3 (x1, y1, z1, x2, y2, z2, ...)
> ```
>
> in which each set of three arguments is treated as a separate line or
> set of lines in three dimensions.
>
> To plot multiple one- or two-argument groups, separate each group
> with an empty format string, as
>
> ```
>           plot3 (x1, c1, "", c2, "", ...)
> ```
>
> An example of the use of `plot3` is
>
> ```
>              z = [0:0.05:5];
>              plot3 (cos(2*pi*z), sin(2*pi*z), z, ";helix;");
>              plot3 (z, exp(2i*pi*z), ";complex sinusoid;");
> ```
>
>
>
>
> **See also:** plot, xlabel, ylabel, zlabel, title, print.

— Function File: **view** (azimuth, elevation)  
— Function File: **view** (dims)  
— Function File: [azimuth, elevation] = **view** ()  
> Set or get the viewpoint for the current axes.

— Function File: **slice** (x, y, z, v, sx, sy, sz)  
— Function File: **slice** (x, y, z, v, xi, yi, zi)  
— Function File: **slice** (v, sx, sy, sz)  
— Function File: **slice** (v, xi, yi, zi)  
— Function File: h = **slice** (...)  
— Function File: h = **slice** (..., method)  
> Plot slices of 3D data/scalar fields. Each element of the 3-dimensional
> array v represents a scalar value at a location given by the
> parameters x, y, and z. The parameters x,
> x, and z are either 3-dimensional arrays of the same size
> as the array v in the "meshgrid" format or vectors. The
> parameters xi, etc. respect a similar format to x, etc.,
> and they represent the points at which the array vi is
> interpolated using interp3. The vectors sx, sy, and
> sz contain points of orthogonal slices of the respective axes.
>
> If x, y, z are omitted, they are assumed to be
> `x = 1:size (`v`, 2)`, `y = 1:size (`v`, 1)` and
> `z = 1:size (`v`, 3)`.
>
> Method is one of:
>
> `"nearest"`: Return the nearest neighbor. `"linear"`: Linear interpolation from nearest neighbors. `"cubic"`: Cubic interpolation from four nearest neighbors (not implemented yet). `"spline"`: Cubic spline interpolation—smooth first and second derivatives throughout the curve.
>
> The default method is `"linear"`.
> The optional return value h is a vector of handles to the
> surface graphic objects.
>
> Examples:
>
> ```
>           [x, y, z] = meshgrid (linspace (-8, 8, 32));
>           v = sin (sqrt (x.^2 + y.^2 + z.^2)) ./ (sqrt (x.^2 + y.^2 + z.^2));
>           slice (x, y, z, v, [], 0, []);
>           [xi, yi] = meshgrid (linspace (-7, 7));
>           zi = xi + yi;
>           slice (x, y, z, v, xi, yi, zi);
> ```
>
>
>
>
> **See also:** interp3, surface, pcolor.

— Function File: **ribbon** (x, y, width)  
— Function File: **ribbon** (y)  
— Function File: h = **ribbon** (...)  
> Plot a ribbon plot for the columns of y vs. x. The
> optional parameter width specifies the width of a single ribbon
> (default is 0.75). If x is omitted, a vector containing the
> row numbers is assumed (1:rows(Y)). If requested, return a vector
> h of the handles to the surface objects.
>
>
>
> **See also:** gca, colorbar.

— Function File: **shading** (type)  
— Function File: **shading** (ax, ...)  
> Set the shading of surface or patch graphic objects. Valid arguments
> for type are
>
> `"flat"`: Single colored patches with invisible edges. `"faceted"`: Single colored patches with visible edges. `"interp"`: Color between patch vertices are interpolated and the patch edges are invisible.
>
> If ax is given the shading is applied to axis ax instead
> of the current axis.

- Three-dimensional Function Plotting- Three-dimensional Geometric Shapes
